# Evaluation on the diagnostic and prognostic values of long non-coding RNA BLACAT1 in common types of human cancer

**DOI:** 10.1186/s12943-017-0728-2

**Published:** 2017-10-16

**Authors:** Xiaoli Chen, Meiyu Dai, Hongzhen Zhu, Jinwan Li, Zhizhuo Huang, Xuexiang Liu, Yujie Huang, Jingfan Chen, Shengming Dai

**Affiliations:** 1 Medical Science Laboratory, the Fourth Affiliated Hospital of Guangxi Medical University, Liuzhou, Guangxi 545005 China; 2 Department of General Surgery, the Fourth Affiliated Hospital of Guangxi Medical University, Liuzhou, Guangxi 545005 China; 3 No.1 LIUSHI Rd, Liuzhou city, Guangxi Province 545005 China

**Keywords:** Long non-coding RNA, BLACAT1, Cancer, TCGA, Diagnosis, Prognosis

## Abstract

A growing number of evidence has indicated that long non-coding RNAs (lncRNA) may have many functions in the development and progression of cancer, and cloud serve as good diagnostic and prognostic biomarkers in cancers. However, these studies often revealed the changes of lncRNAs within a specific cancer type. Here, we focused on BLACAT1 and provided a comprehensive pan-cancer analysis to evaluate the diagnostic and prognostic values of BLACAT1. The expression data of BLACAT1 were came from the quantitative real-time polymerase chain reaction (qRT-PCR) and The Cancer Genome Atlas (TCGA) database, respectively. Our results showed that the change of serum BLACAT1 expression was similar to those in matched tissues. The expression level of BLACAT1 both in serum and tissues in multiple cancer types were significantly upregulated compared to those of matched non-cancer participants. The serum BLACAT1 had a high diagnostic performance among these 12 types of cancer. The relative AUC of serum BLACAT1 in cancer patients ranged from 0.833 to 0.967 compared to that in healthy subjects. Surprisingly, Kaplan-Meier survival analysis revealed that the high expression level of BLACAT1 was significantly associated with poor overall survival only in uterine corpus endometrial carcinoma (*p* = 0.002, log-rank test). These findings demonstrated that BLACAT1 could act as a non-specific diagnostic biomarker for cancers and a potential biomarker for prognosis prediction of endometrial cancer.

**Electronic supplementary material:**

The online version of this article (10.1186/s12943-017-0728-2) contains supplementary material, which is available to authorized users.

## Background

Although the advance in surgical techniques and chemoradiotherapy, cancer is still one of the diseases that threaten human beings’ health and lives seriously. Blood-based tumor markers have always been a hot spot of research for diagnostic and prognostic markers as they are noninvasive and highly reproducible at low cost. Many molecular markers have been reported to predict the occurrence and treatment of cancer [1–3]. However, no specific or sensitive biomarkers have been confirmed and used in clinical practice to predict the occurrence and outcome of cancer up to now. According to the systematically calculation from American Cancer Society, the sensitivity of a prostatic specific antigen cutoff of 4.0 ng/mL was 21% for detecting any prostate cancer (PC) and 51% for detecting high-grade cancers (Gleason ≥8) [4]. Liu et al. had reported that the sensitivity and specificity of the diagnostic marker carcinoembryonic antigen for colorectal cancer (CRC) were 46.1% and 89.2%, respectively [5]. Serum alpha-fetoprotein, as a gold standard in hepatocellular carcinoma (HCC) detection, has low diagnostic accuracy, with sensitivities ranging from a mere 18–60% and a specificity of ~85–90% [6].

Long non-coding RNAs (lncRNAs) have many functions in various pathophysiological processes [7–10]. The dysregulation of lncRNAs plays pivotal roles in many kinds of diseases, particularly in cancer [11–13]. The lncRNAs circulating in serum/plasma are relatively stable because they are not degraded by RNase even in the complex environment in vivo [14]. Thus, the lncRNAs could be acted as potential diagnostic or prognostic markers in multiple types of cancer [15–18]. BLACAT1, known as linc-UBC1, is found on human chromosome 1q32.1 and has a transcript of 2616 kb with just one exon [19]. It was firstly reported as a negative prognostic factor for lymph node metastasis and survival in bladder cancer (BLC) [20]. Our previous research has shown that BLACAT1 was upregulated in CRC tissues compared to adjacent normal tissues and could serve as a novel diagnostic biomarker [21]. However, these studies have just explored the clinical and biological significance in a specific cancer type. Thus, the diagnostic and prognostic values of BLACAT1 in many other cancer types are still unclear.

In this study, we evaluated the diagnostic value of serum BLACAT1 across 12 common types of cancers, and used RNA-seq datasets of The Cancer Genome Atlas (TCGA) to evaluate the prognostic value of BLACAT1 in 14 types of cancers. Our results showed that BLACAT1 could serve as a non-specific diagnostic marker for these types of cancer and prognostic marker only in endometrial cancer (EMC).

## Result

### Serum BLACAT1 could be served as a non-specific biomarker for the diagnosis in cancer

**Fig. 1:**
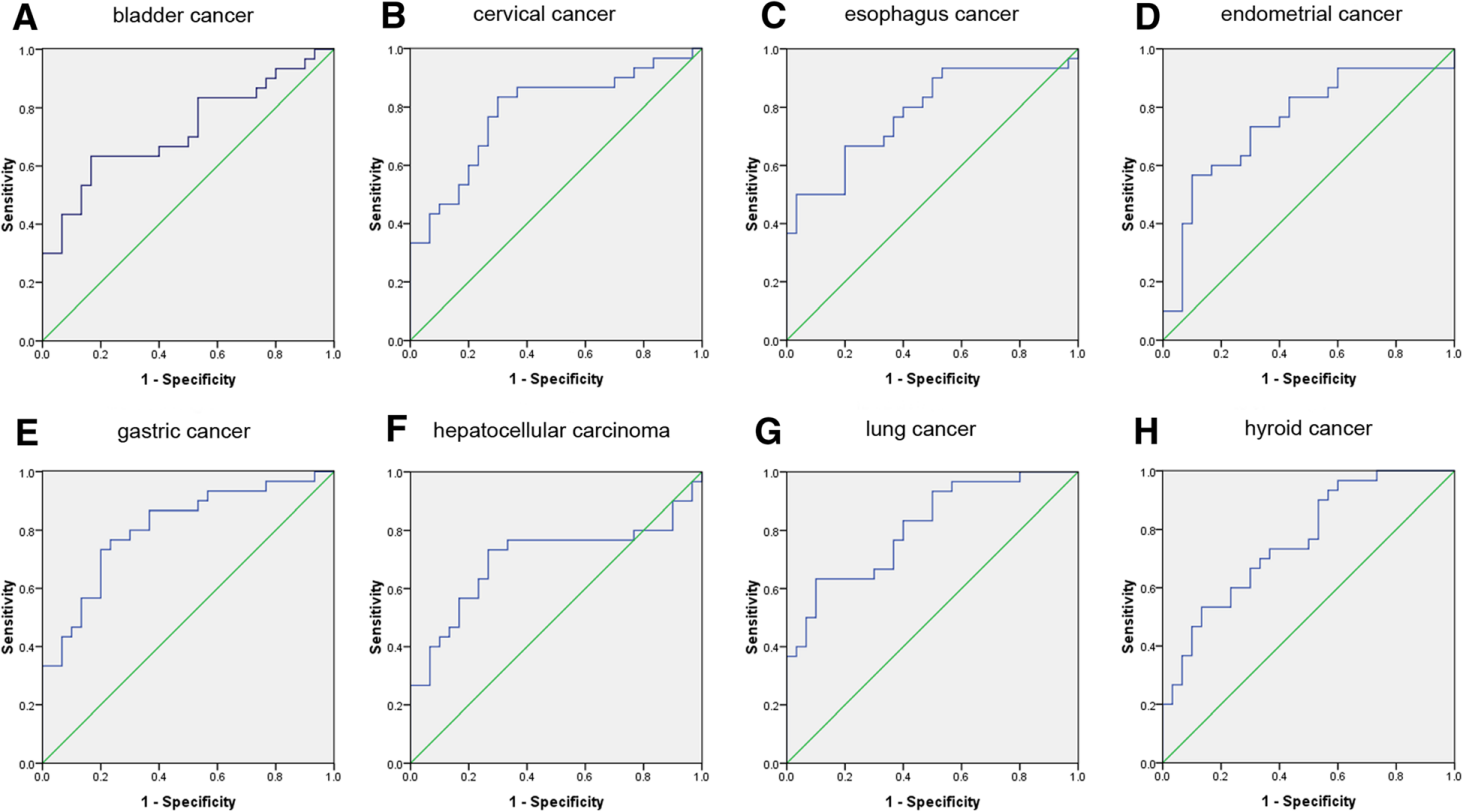
The ROC analyses for serum BLACAT1 in discriminating 8 types of cancer from matched benign lesion. (**a**) bladder cancer; (**b**) cervical cancer; (**c**) esophagus cancer; (**d**) endometrial cancer; (**e**) gastric cancer; (**f**) hepatocellular carcinoma; (**g**) lung cancer; (**h**) hyroid cancer

To test whether BLACAT1 was a specific biomarker for a specific cancer type, we used the qRT-PCR to measure 1080 serum samples in 12 common types of cancer and the corresponding benign lesions and healthy subjects (Additional file 1: Table S1). These 12 different types of cancer included: HCC, lung cancer (LC), breast cancer (BC), ovarian cancer (OC), EMC, cervical cancer (CC), PC, gastric cancer (GC), esophagus cancer (EC), thyroid cancer (TC), BLC and nasopharynx cancer (NPC). For the diagnostic value of serum BLACAT1 in CRC was reported in our previous article, the CRC was not included in present study [21]. According to the qRT-PCR confirmation results, serum BLACAT1 was performed well in distinguishing cancer from healthy subjects in these 12 types of cancer, which was not able to distinguish cancer from benign lesions in BC, OC, PC and NPC (Additional file 1: Figure S1). To further investigate the diagnostic performance of serum BLACAT1, we then performed an ROC curve analysis. With significantly differentiated level in cancer subjects as compared with healthy subjects, the relative AUC of serum BLACAT1 in these 12 types of cancer was from 0.833 to 0.967 (Fig. 1). The diagnostic values of serum BLACAT1 in 12 types of cancer were summered in Table 1.

**Table 1:** BLACAT1 could act as a non-specific diagnostic biomarker for these common cancers

variable	cancer vs benign			cancer vs healthy			benign vs healthy		
	*p*-value	FC	AUC	*p*-value	FC	AUC	*p*-value	FC	AUC
hepatocellular carcinoma	0.01	2.4	0.7	<0.01	5.3	0.878	<0.01	2.2	0.891
lung cancer	<0.01	2.8	0.81	<0.01	5.3	0.967	0.06	1.9	–
breast cancer	<0.01	1.8	–	<0.01	2.9	0.864	0.04	1.6	–
ovarian cancer	<0.01	1.6	–	<0.01	3.1	0.933	<0.01	1.9	–
endometrial cancer	<0.01	2.2	0.751	<0.01	6.6	0.932	<0.01	2.9	0.987
cervical cancer	<0.01	2.6	0.782	<0.01	5.9	0.963	<0.01	2.3	0.891
prostate cancer	0.111	1.6	–	<0.01	4.1	0.833	<0.01	2.6	0.827
gastric cancer	<0.01	2.6	0.808	<0.01	4.5	0.933	0.02	1.7	–
esophagus cancer	<0.01	2.7	0.781	<0.01	6.2	0.933	<0.01	2.3	0.966
hyroid cancer	<0.01	2.1	0.762	<0.01	2.9	0.933	0.76	1.4	–
bladder cancer	<0.01	2.9	0.722	<0.01	4.7	0.833	0.047	1.6	–
nasopharynx cancer	0.01	1.6	–	<0.01	2.4	0.867	<0.01	1.5	–

*FC*: fold-change; *p*: *p*-value by students’ *T*-test; *AUC*: Area under ROC curve

### Evaluated the prognostic value of BLACAT1 in 14 types of cancer based on TCGA database

**Fig. 2:**
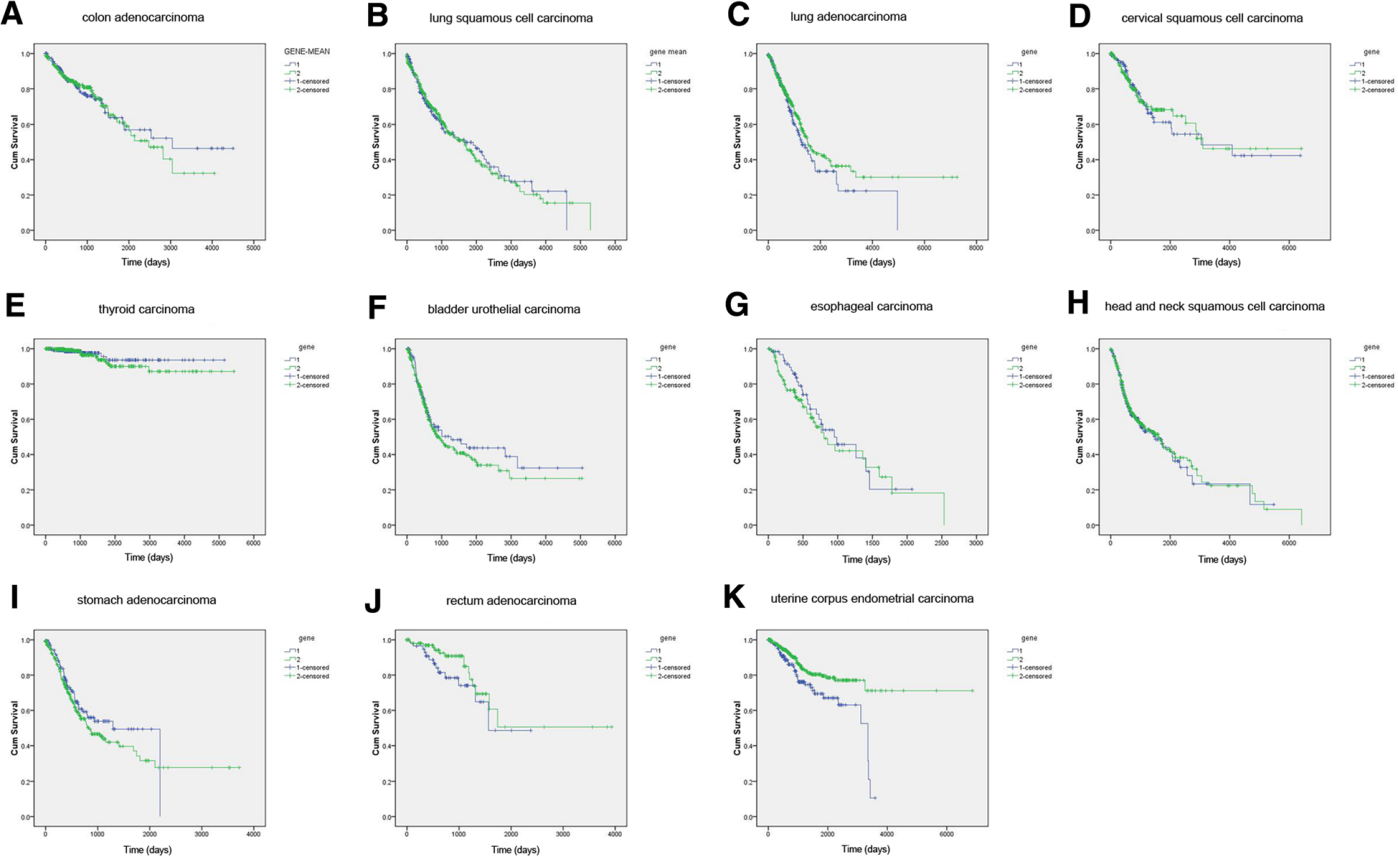
Kaplan-Meier survival curves for 11 cancer types. The expression values of BLACAT1 were classified into high or low BLACAT1 expression groups according to the mean expression level. 1 represented high expression group. 2 represented low expression group. (**a**) colon adenocarcinoma; (**b**) lung squamous cell carcinoma; (**c**) lung adenocarcinoma; (**d**) cervical squamous cell carcinoma; (**e**) thyroid carcinoma; (**f**) bladder urothelial carcinoma; (**g**) esophageal carcinoma; (**h**) head and neck squamous cell carcinoma; (**i**) stomach adenocarcinoma; (**j**) rectum adenocarcinoma; (**k**) uterine corpus endometrial carcinoma. The OS of uterine corpus endometrial carcinoma patients with a high expression of BLACAT1 decreased significantly compared with those with a low level of BLACAT1 (*p*=0.002, log-rank test)

Based on the TCGA database, we could conduct a pan-cancer analysis to evaluate the relative expression level of BLACAT1 in the 14 types of cancer between cancer tissues and adjacent normal tissues (Additional file 1: Table S2). The 14 common types of cancer were downloaded from TCGA: breast invasive carcinoma (BRCA); lung adenocarcinoma (LUAD); uterine corpus endometrial carcinoma (UCEC); head and neck squamous cell carcinoma (HNSC); thyroid carcinoma (THCA); lung squamous cell carcinoma (LUSC); prostate adenocarcinoma (PRAD); colon adenocarcinoma (COAD); stomach adenocarcinoma (STAD); bladder urothelial carcinoma (BLCA); liver hepatocellular carcinoma (LIHC); cervical squamous cell carcinoma (CESC); esophageal carcinoma (ESCA); rectum adenocarcinoma (READ). The LIHC and PRAD were removed from our study for the missing value greater than 10%. As shown in Additional file 1: Figure S2, the change of BLACAT1 expression in serum was similar in matched tissues. The BRCA had not significant difference in the mean expression value of BLACAT1 (fold-change = 1.29, *p*-value = 8.44E-06). The expression level of BLACAT1 in COAD was consistent with our previous study [18]. Meanwhile, we found that high expression of BLACAT1 was associated with advanced TNM staging in COAD, READ and THCA, indicating that BLACAT1 might be an oncogene in these types of cancer (Additional file 1: Table S3). We also found, unexpectedly, that the gender also exhibited a significant correlation with high expression BLACAT1 in COAD and HNSC (*p* = 0.01 and 0.034, respectively). These results could provide us a new and useful reference to explain the difference in prognosis between male and female with high expression of BLACAT1 in COAD and HNSC. These data encouraged us to explore the prognostic value of BLACAT1 expression in these types of cancer. Surprisingly, Kaplan-Meier survival analysis revealed that there was no statistical difference among these cancers except the UCEC (Fig. 2). The OS of UCEC patients with a high expression of BLACAT1 decreased significantly compared with those with a low level of BLACAT1 (*p* = 0.002, log-rank test). This result suggested that high BLACAT1 expression could be regarded as a specific prognostic factor In UCEC. This result was inconsistent with the previous studies that reported the BLACAT1 could act as a prognostic factor in GC, BLC and CRC [19–21]. One possible reason was that normal tissues were fewer than cancer tissues in TCGA database. For instance, there are only three normal tissues in cervical squamous cell carcinoma. Therefore, this result needed further investigation in a larger patient cohort.

### Functional enrichment analysis of BLACAT1 and its related genes

To explore the potential mechanisms of BLACAT1 in cancer, the Multi-Experiment Matrix (MEM) was used to distinguish genes related to BLACAT1. The top 100 genes were selected for GO and KEGG pathway analyses (Additional file 1: Figure S3). As our results showed, these genes were involved in many different biological processes and molecular functions (Additional file 1: Figure S4). However, only one KEGG pathways (the Hippo signaling pathway) were significant in our study. The Hippo signaling pathway is an evolutionarily conserved kinase cascade involved in organ size control, tissue homeostasis and cancer [22, 23]. Previous reports suggested that the Hippo signaling pathway was involved in EMC tumorigenesis and correlated with a poor prognosis [24–26]. The consistent result was also found in our study.

## Conclusion

We provided a comprehensive pan-cancer analysis of BLACAT1 expression based on serum samples and the TCGA data in multiple cancer types. Our results demonstrated that BLACAT1 increased with non-specificity in cancer tissues and serum compared with healthy controls. In this sense, BLACAT1 could act as a non-specific diagnostic biomarker for cancer and potential biomarker for prognosis prediction of EMC.

## Additional file

Additional file 1:
**Table S1.**The clinical features of the patients. **Table S2.** Clinical features of the patients from TCGA database. **Table S3.** The correlation analysis between BLACAT1 expression and clinicopathologic factors in different types of cancer. **Figure S1.** The expression levels of BLACAT1 in 12 types of cancer patients and its comparison with those in the serum of matched non-cancer participants. **Figure S2.** Expression levels of BLACAT1 in 12 types of cancer tissues and normal tissues. The black horizontal lines were median values with standard deviation. The *p* values were determined by the two-tailed Student’s *t*-test. **Figure S3.** Gene network of the related genes of BLACAT1. BLACAT1 was shown as yellow node, and the related genes were shown as red nodes. The related genes were enriched by the MEM. The top 100 genes were selected for further analysis. **Figure S4.** The enriched GO items (biological process and molecular function) with BLACAT1-related genes. (DOCX 26265 kb)

## Abbreviations

AUC: Area under ROC curve
BC: Breast cancer
BLACAT1: Bladder cancer associated transcript 1
BLC: Bladder cancer
BLCA: Bladder urothelial carcinoma
BRCA: Breast invasive carcinoma
CC: Cervical cancer
CESC: Cervical squamous cell carcinoma
COAD: Colon adenocarcinoma
CRC: Colorectal cancer
EC: Esophagus cancer
EMC: Endometrial cancer
ESCA: Esophageal carcinoma
GC: Gastric cancer
GO: Gene Ontology
HC: Healthy control
HCC: Hepatocellular carcinoma
HNSC: Head and neck squamous cell carcinoma
KEGG: Kyoto Encyclopedia of Genes and Genomes
LC: Lung cancer
LIHC: Liver hepatocellular carcinoma
lncRNA: Long non-coding RNAs
LUAD: Lung adenocarcinoma
LUSC: Lung squamous cell carcinoma
MEM: Multi Experiment Matrix
NPC: Nasopharynx cancer
OC: Ovarian cancer
OS: Overall survival
PC: Prostate cancer
PRAD: Prostate adenocarcinoma
qRT-PCR: quantitative real-time polymerase chain reaction
READ: Rectum adenocarcinoma
ROC: Receiver operating characteristic
STAD: Stomach adenocarcinoma
TC: Hyroid cancer
TCGA: The Cancer Genome Atlas
THCA: Thyroid carcinoma
UCEC: Uterine corpus endometrial carcinoma

## References

[1] Obort AS, Ajadi MB, Akinloye O (2013). Prostate-specific antigen: any successor in sight?. Rev Urol.

[2] Liu L, Zhao Y, Jia J, Chen H, Bai W, Yang M, Yin Z, He C, Zhang L, Guo W (2016). The Prognostic Value of Alpha-Fetoprotein Response for Advanced-Stage Hepatocellular Carcinoma Treated with Sorafenib Combined with Transarterial Chemoembolization. Sci Rep.

[3] Jain P, Mondal SK, Sinha SK, Mukhopadhyay M, Chakraborty I (2014). Diagnostic and prognostic significance of different mucin expression, preoperative CEA, and CA-125 in colorectal carcinoma: A clinicopathological study. J Nat Sci Biol Med.

[4] Wolf AM, Wender RC, Etzioni RB, Thompson IM, D'Amico AV, Volk RJ, Brooks DD, Dash C, Guessous I, Andrews K (2010). American Cancer Society guideline for the early detection of prostate cancer: update 2010. CA Cancer J Clin.

[5] Liu Z, Zhang Y, Niu Y, Li K, Liu X, Chen H, Gao C (2014). A systematic review and meta-analysis of diagnostic and prognostic serum biomarkers of colorectal cancer. PLoS One.

[6] Reichl P, Mikulits W (2016). Accuracy of novel diagnostic biomarkers for hepatocellular carcinoma: An update for clinicians (Review). Oncol Rep.

[7] Nagano T, Fraser P (2011). No-nonsense functions for long noncoding RNAs. Cell.

[8] Ponting CP, Oliver PL, Reik W (2009). Evolution and functions of long noncoding RNAs. Cell.

[9] Kugel JF, Goodrich JA (2012). Non-coding RNAs: key regulators of mammalian transcription. Trends Biochem Sci.

[10] Prensner JR, Chen W, Iyer MK, Cao Q, Ma T, Han S, Sahu A, Malik R, Wilder-Romans K, Navone N (2014). PCAT-1, a long noncoding RNA, regulates BRCA2 and controls homologous recombination in cancer. Cancer Res.

[11] Iyer MK, Niknafs YS, Malik R, Singhal U, Sahu A, Hosono Y, Barrette TR, Prensner JR, Evans JR, Zhao S (2015). The landscape of long noncoding RNAs in the human transcriptome. Nat Genet.

[12] Tang L, Zhang W, Su B, Yu B (2013). Long noncoding RNA HOTAIR is associated with motility, invasion, and metastatic potential of metastatic melanoma. Biomed Res Int.

[13] Ge XS, Ma HJ, Zheng XH, Ruan HL, Liao XY, Xue WQ, Chen YB, Zhang Y, Jia WH (2013). HOTAIR, a prognostic factor in esophageal squamous cell carcinoma, inhibits WIF-1 expression and activates Wnt pathway. Cancer Sci.

[14] Zhang X, Zhang W, Cao WD, Cheng G, Zhang YQ (2012). Glioblastoma multiforme: Molecular characterization and current treatment strategy (Review). Exp Ther Med.

[15] Zheng S, Zheng D, Dong C, Jiang J, Xie J, Sun Y, Chen H. Development of a novel prognostic signature of long non-coding RNAs in lung adenocarcinoma. J Cancer Res Clin Oncol. 2017; 10.1007/s00432-017-2411-9 PMC11818973 28409273

[16] He B, Zeng J, Chao W, Chen X, Huang Y, Deng K, Huang Z, Li J, Dai M, Chen S (2017). Serum long non-coding RNAs MALAT1, AFAP1-AS1 and AL359062 as diagnostic and prognostic biomarkers for nasopharyngeal carcinoma. Oncotarget.

[17] Li X, Wang F, Sun Y, Fan Q, Cui G (2017). Expression of long non-coding RNA PANDAR and its prognostic value in colorectal cancer patients. Int J Biol Markers.

[18] Nie ZL, Wang YS, Mei YP, Lin X, Zhang GX, Sun HL, Wang YL, Xia YX, Wang SK. Prognostic significance of long noncoding RNA Z38 as a candidate biomarker in breast cancer. J Clin Lab Anal. 2017; 10.1002/jcla.22193 PMC6816943 28247935

[19] Hu Y, Pan J, Wang Y, Li L, Huang Y (2015). Long noncoding RNA linc-UBC1 is negative prognostic factor and exhibits tumor pro-oncogenic activity in gastric cancer. Int J Clin Exp Pathol.

[20] He W, Cai Q, Sun F, Zhong G, Wang P, Liu H, Luo J, Yu H, Huang J, Lin T (2013). Linc-UBC1 physically associates with polycomb repressive complex 2 (PRC2) and acts as a negative prognostic factor for lymph node metastasis and survival in bladder cancer. Biochim Biophys Acta.

[21] Dai M, Chen X, Mo S, Li J, Huang Z, Huang S, Xu J, He B, Zou Y, Chen J, Dai S (2017). Meta-signature LncRNAs serve as novel biomarkers for colorectal cancer: integrated bioinformatics analysis, experimental validation and diagnostic evaluation. Sci Rep.

[22] Harvey KF, Zhang X, Thomas DM (2013). The Hippo pathway and human cancer. Nat Rev Cancer.

[23] Pan D (2010). The hippo signaling pathway in development and cancer. Dev Cell.

[24] Mitamura T, Watari H, Wang L, Kanno H, Kitagawa M, Hassan MK, Kimura T, Tanino M, Nishihara H, Tanaka S, Sakuragi N (2014). microRNA 31 functions as an endometrial cancer oncogene by suppressing Hippo tumor suppressor pathway. Mol Cancer.

[25] Romero-Perez L, Garcia-Sanz P, Mota A, Leskela S, Hergueta-Redondo M, Diaz-Martin J, Lopez-Garcia MA, Castilla MA, Martinez-Ramirez A, Soslow RA (2015). A role for the transducer of the Hippo pathway, TAZ, in the development of aggressive types of endometrial cancer. Mod Pathol.

[26] Wang C, Jeong K, Jiang H, Guo W, Gu C, Lu Y, Liang J (2016). YAP/TAZ regulates the insulin signaling via IRS1/2 in endometrial cancer. Am J Cancer Res.

