# Supplementary material for: Evaluation on the diagnostic and prognostic values of long non-coding RNA BLACAT1 in common types of human cancer
Source: Mol Cancer. 2017 Oct 16;16:160. doi: 10.1186/s12943-017-0728-2 (PMC5644079; doi:10.1186/s12943-017-0728-2)
Supplement: Additional file 1: — Table S1.The clinical features of the patients. Table S2. Clinical features of the patients from TCGA database. Table S3. The correlation analysis between BLACAT1 expression and clinicopathologic factors in different types of cancer. Figure S1. The expression levels of BLACAT1 in 12 types of cancer patients and its comparison with those in the serum of matched non-cancer participants. Figure S2. Expression levels of BLACAT1 in 12 types of cancer tissues and normal tissues. The black horizontal lines were median values with standard deviation. The p values were determined by the two-tailed Student’s t-test. Figure S3. Gene network of the related genes of BLACAT1. BLACAT1 was shown as yellow node, and the related genes were shown as red nodes. The related genes were enriched by the MEM. The top 100 genes were selected for further analysis. Figure S4. The enriched GO items (biological process and molecular function) with BLACAT1-related genes. (DOCX 26265 kb) [file 12943_2017_728_MOESM1_ESM.docx]

Table S1. The clinical features of the patients.

| variable | GC | CLG | HC | HCC | CHB | HC | LC | COPD | HC | BC | GT | HC | CC | CIN | HC | OC | ORC | HC | EMC | fibroid | HC | PC | CP | HC | EC | OES | HC | TC | THY | HC | BLC | URO | HC | NPC | NAS | HC |
| --- | --- | --- | --- | --- | --- | --- | --- | --- | --- | --- | --- | --- | --- | --- | --- | --- | --- | --- | --- | --- | --- | --- | --- | --- | --- | --- | --- | --- | --- | --- | --- | --- | --- | --- | --- | --- |
| number | 30 | 30 | 30 | 30 | 30 | 30 | 30 | 30 | 30 | 30 | 30 | 30 | 30 | 30 | 30 | 30 | 30 | 30 | 30 | 30 | 30 | 30 | 30 | 30 | 30 | 30 | 30 | 30 | 30 | 30 | 30 | 30 | 30 | 30 | 30 | 30 |
| gender |  |  |  |  |  |  |  |  |  |  |  |  |  |  |  |  |  |  |  |  |  |  |  |  |  |  |  |  |  |  |  |  |  |  |  |  |
| male | 18 | 18 | 18 | 27 | 27 | 27 | 18 | 18 | 18 | - | - | - | - | - | - | - | - | - | - | - | - | 30 | 30 | 30 | 30 | 30 | 30 | 6 | 6 | 6 | 24 | 24 | 24 | 15 | 15 | 15 |
| female | 12 | 12 | 12 | 3 | 3 | 3 | 12 | 12 | 12 | 30 | 30 | 30 | 30 | 30 | 30 | 30 | 30 | 30 | 30 | 30 | 30 | - | - | - | - | - | - | 24 | 24 | 24 | 6 | 6 | 6 | 15 | 15 | 15 |
| Age  (years) | 57.6 | 57.5 | 58.1 | 54.5 | 52.4 | 52.8 | 59.7 | 59.1 | 59.6 | 45.6 | 45.7 | 46.2 | 49.7 | 48.1 | 49.3 | 48.1 | 48.1 | 48.8 | 49.6 | 50 | 50.3 | 62.4 | 62 | 61.2 | 59.4 | 59.1 | 58.5 | 50.9 | 48.1 | 49.5 | 59.7 | 61 | 60.2 | 49.2 | 50.1 | 51.1 |
| stage |  |  |  |  |  |  |  |  |  |  |  |  |  |  |  |  |  |  |  |  |  |  |  |  |  |  |  |  |  |  |  |  |  |  |  |  |
| I/II | 10 | - | - | 12 | - | - | 13 | - | - | 15 | - | - | 13 | - | - | 16 | - | - | 14 | - | - | 8 | - | - | 6 | - | - | 7 | - | - | 11 | - | - | 13 | - | - |
| III/IV | 20 | - | - | 18 | - | - | 17 | - | - | 15 | - | - | 17 | - | - | 14 | - | - | 16 | - | - | 22 | - | - | 24 | - | - | 23 | - | - | 19 | - | - | 17 | - | - |
| HCC: hepatocellular carcinoma; LC: lung cancer; BC: breast cancer; OC: ovarian cancer; EMC: endometrial cancer; CC: cervical cancer; PC: prostate cancer; GC: gastric cancer; EC: esophagus cancer; TC: hyroid cancer; BLC: bladder cancer; NPC: nasopharynx cancer; CLG: chronic gastritis; COPD: chronic obstructive pulmonary disease; GT: galactoma; ORC: ovarian cysts; CIN: cervical Intraepithelial Neoplasia; CP: chronic prostatitis; OES: oesophagitis; THY: thyrocele; URO: urocystitis; NAS: nasopharyngitis; CHB: chronic viral hepatitis b; HC: healthy control. | | | | | | | | | | | | | | | | | | | | | | | | | | | | | | | | | | | | |

Table S2. Clinical features of the patients from TCGA database.

| variable | BRCA | normal |  | LUAD | normal |  | UCEC | normal |  | HNSC | normal |  | THCA | normal |  | LUSC | normal |  | PRAD | normal |  | COAD | normal |  | STAD | normal |  | BLCA | normal |  | LIHC | normal |  | CESC | normal |  | ESCA | normal |  | READ | normal |
| --- | --- | --- | --- | --- | --- | --- | --- | --- | --- | --- | --- | --- | --- | --- | --- | --- | --- | --- | --- | --- | --- | --- | --- | --- | --- | --- | --- | --- | --- | --- | --- | --- | --- | --- | --- | --- | --- | --- | --- | --- | --- |
| number | 1108 | 113 |  | 502 | 49 |  | 552 | 23 |  | 502 | 44 |  | 510 | 58 |  | 535 | 59 |  | 499 | 52 |  | 478 | 41 |  | 375 | 32 |  | 414 | 19 |  | 478 | 41 |  | 303 | 3 |  | 162 | 11 |  | 164 | 10 |
| gender |  |  |  |  |  |  |  |  |  |  |  |  |  |  |  |  |  |  |  |  |  |  |  |  |  |  |  |  |  |  |  |  |  |  |  |  |  |  |  |  |  |
| male | - | - |  | 371 | 35 |  | - | - |  | 368 | 30 |  | 139 | 17 |  | 249 | 25 |  | 499 | 52 |  | 252 | 20 |  | 241 | 22 |  | 305 | 10 |  | 252 | 20 |  | - | - |  | 139 | 8 |  | 89 | 3 |
| female | 1108 | 113 |  | 131 | 14 |  | 552 | 23 |  | 134 | 14 |  | 371 | 41 |  | 286 | 34 |  | - | - |  | 226 | 21 |  | 134 | 10 |  | 109 | 9 |  | 226 | 21 |  | 303 | 3 |  | 23 | 3 |  | 75 | 7 |
| Age(years) | 58.3 | 57.3 |  | 66.0 | 68.6 |  | 64.0 | 59.5 |  | 61.0 | 62.1 |  | 47.3 | 45.6 |  | 62.7 | 66.1 |  | 61.0 | 60.3 |  | 67.0 | 70.3 |  | 65.1 | 68.8 |  | 68.0 | 69.9 |  | 67.0 | 70.3 |  | 48.0 | 54.3 |  | 62.2 | 70.3 |  | 64.7 | 63.8 |
| stage |  |  |  |  |  |  |  |  |  |  |  |  |  |  |  |  |  |  |  |  |  |  |  |  |  |  |  |  |  |  |  |  |  |  |  |  |  |  |  |  |  |
| I/II | 814 | 85 |  | 409 | 43 |  | 384 | 14 |  | 283 | 11 |  | 338 | 42 |  | 423 | 44 |  | - | - |  | 276 | 27 |  | 164 | 21 |  | 134 | 4 |  | 268 | 26 |  | 230 | 3 |  | 87 | 7 |  | 84 | 6 |
| III/IV | 283 | 27 |  | 93 | 6 |  | 156 | 9 |  | 219 | 33 |  | 170 | 16 |  | 111 | 15 |  | - | - |  | 202 | 14 |  | 188 | 11 |  | 278 | 15 |  | 199 | 14 |  | 67 | 0 |  | 75 | 4 |  | 80 | 4 |
| BRCA: breast invasive carcinoma; LUAD: lung adenocarcinoma; UCEC: uterine corpus endometrial carcinoma; HNSC: head and neck squamous cell carcinoma; THCA: thyroid carcinoma; LUSC: lung squamous cell carcinoma; PRAD: prostate adenocarcinoma; COAD: colon adenocarcinoma; STAD: stomach adenocarcinoma; BLCA: bladder urothelial carcinoma; LIHC: liver hepatocellular carcinoma; CESC: cervical squamous cell carcinoma; ESCA: esophageal carcinoma; READ: rectum adenocarcinoma. | | | | | | | | | | | | | | | | | | | | | | | | | | | | | | | | | | | | | | | | | |

Table S3. The correlation analysis between BLACAT1 expression and clinicopathologic factors in different types of cancer.

| clinicopathologic factors | COAD | |  | READ | |  | LUAD | |  | LUSC | |  | UCEC | |  | HNSC | |  | THCA | |  | STAD | |  | BLCA | |  | CESC | |  | ESCA | |
| --- | --- | --- | --- | --- | --- | --- | --- | --- | --- | --- | --- | --- | --- | --- | --- | --- | --- | --- | --- | --- | --- | --- | --- | --- | --- | --- | --- | --- | --- | --- | --- | --- |
|  | P value | r |  | P value | r |  | P value | r |  | P value | r |  | P value | r |  | P value | r |  | P value | r |  | P value | r |  | P value | r |  | P value | r |  | P value | r |
| Age | 0.615 | -0.022 |  | 0.716 | -0.028 |  | 0.611 | -0.021 |  | 0.049 | 0.084 |  | 0.121 | 0.065 |  | 0.721 | 0.015 |  | 0.845 | -0.008 |  | 0.877 | 0.008 |  | 0.171 | -0.066 |  | 0.662 | -0.025 |  | 0.741 | -0.025 |
| Gender | **0.01** | -0.112 |  | 0.206 | -0.096 |  | 0.77 | -0.012 |  | 0.324 | -0.042 |  | - | - |  | **0.034** | -0.091 |  | 0.961 | -0.002 |  | 0.23 | -0.06 |  | 0.667 | -0.021 |  | - | - |  | 0.54 | -0.047 |
| TNM staging | **0.001** | 0.142 |  | **0.005** | 0.213 |  | 0.723 | -0.015 |  | 0.948 | 0.003 |  | 0.219 | 0.052 |  | 0.07 | 0.078 |  | **0.033** | 0.089 |  | 0.208 | 0.065 |  | 0.294 | 0.051 |  | 0.845 | -0.011 |  | 0.434 | -0.063 |
| LUAD: lung adenocarcinoma; UCEC: uterine corpus endometrial carcinoma; HNSC: head and neck squamous cell carcinoma; THCA: thyroid carcinoma; LUSC: lung squamous cell carcinoma; COAD: colon adenocarcinoma; STAD: stomach adenocarcinoma; BLCA: bladder urothelial carcinoma; CESC: cervical squamous cell carcinoma; ESCA: esophageal carcinoma; READ: rectum adenocarcinoma. | | | | | | | | | | | | | | | | | | | | | | | | | | | | | | | | |


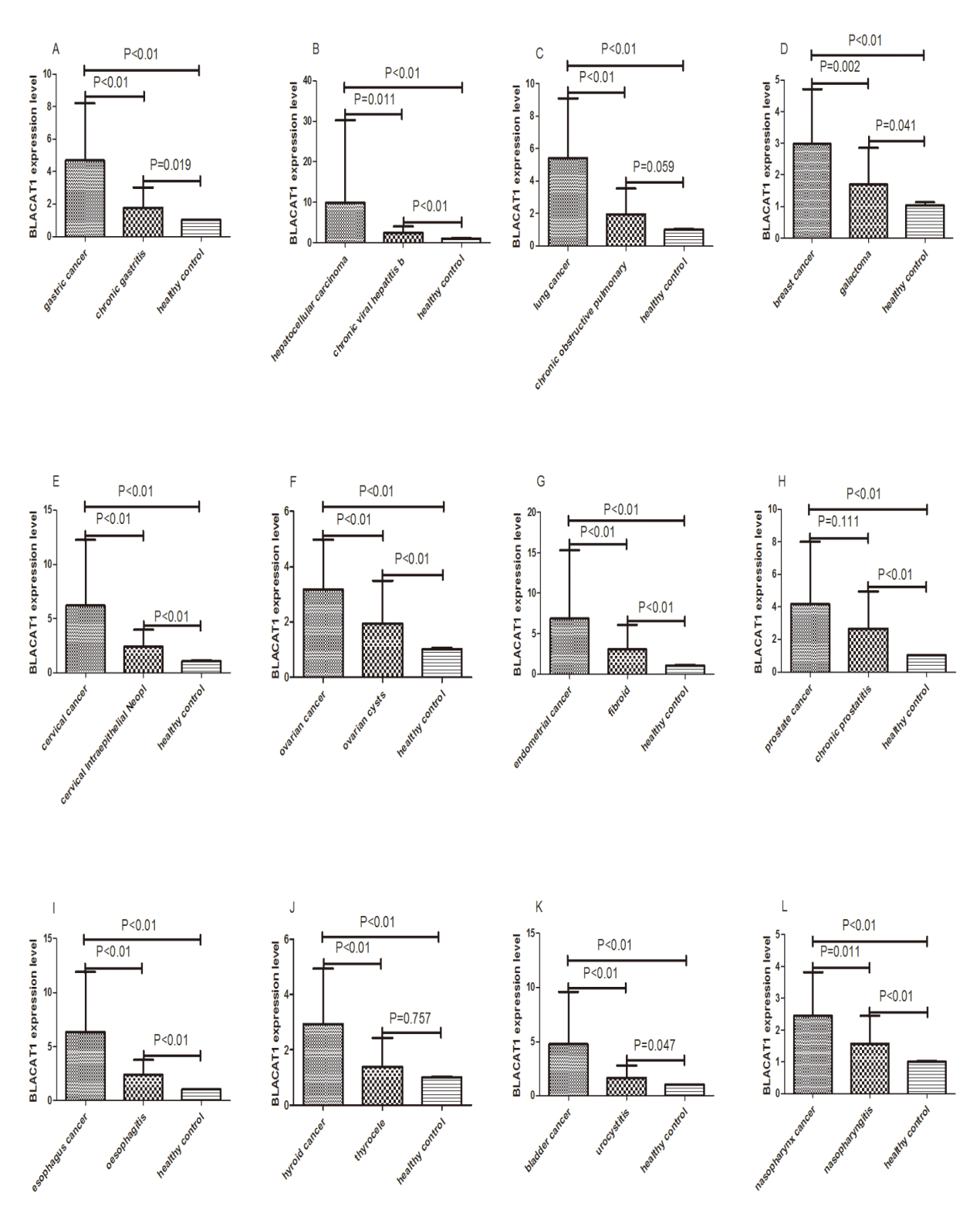


**Figure S1**. The expression levels of BLACAT1 in 12 types of cancer patients and its comparison with those in the serum of matched non-cancer participants.


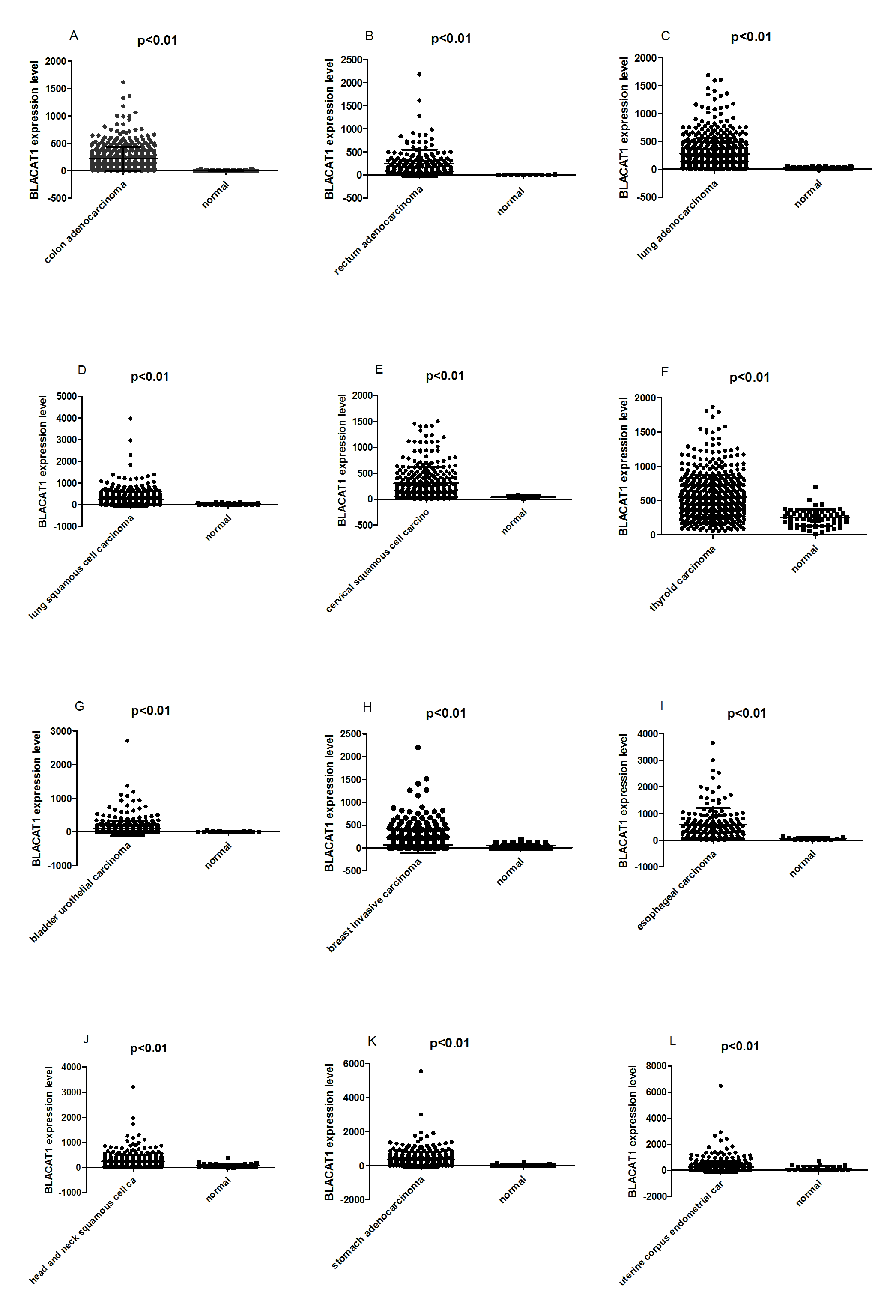


**Figure S2**. Expression levels of BLACAT1 in 12 types of cancer tissues and normal tissues. The black horizontal lines were median values with standard deviation. The *p* values were determined by the two-tailed Student's t-test.


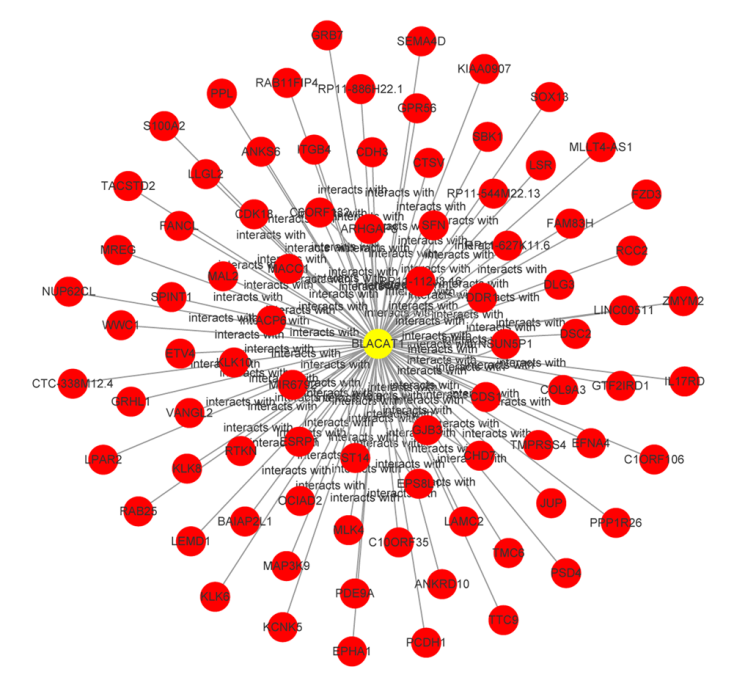


**Figure S3.** Gene network of the related genes of BLACAT1. BLACAT1 was shown as yellow node, and the related genes were shown as red nodes. The related genes were enriched by the MEM. The top 100 genes were selected for further analysis.


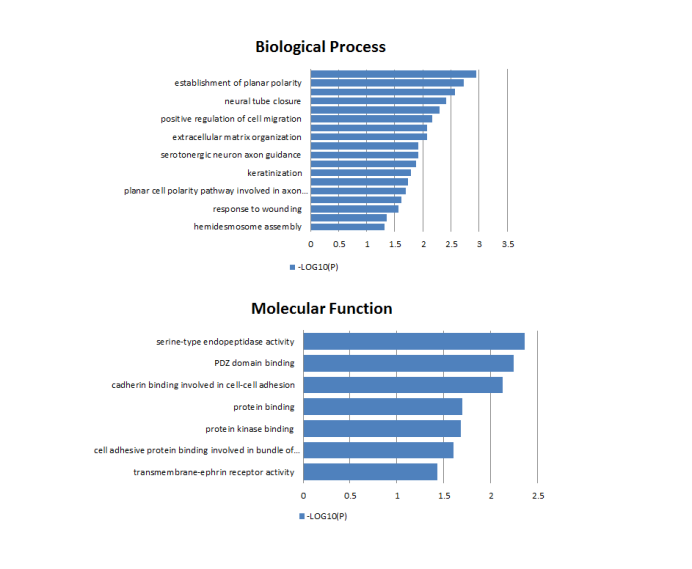


**Figure S4**. The enriched GO items (biological process and molecular function) with BLACAT1-related genes.
